# The Dark Triad and framing effects predict selfish behavior in a one-shot Prisoner’s Dilemma

**DOI:** 10.1371/journal.pone.0203891

**Published:** 2018-09-19

**Authors:** Paul Deutchman, Jessica Sullivan

**Affiliations:** Department of Psychology, Skidmore College, Saratoga Springs, New York, United States of America; Universitat Jaume I, SPAIN

## Abstract

What causes us to display selfish behaviors? We explored the extent to which Dark Triad traits (sub-clinical psychopathy, narcissism, and Machiavellianism) support a selfish behavioral strategy. We related performance on a hypothetical Prisoner’s Dilemma (an economic game that simulates a two-person social dilemma) to participants’ (*N* = 1400) Dark Triad scores. Because contextual factors also impact selfish behaviors, we tested how framing (gain vs. loss; and social vs. nonsocial) shaped performance in the Prisoner’s Dilemma. Participants with high Dark Triad scores were more likely to behave selfishly in the Prisoner’s Dilemma. Participants were also most likely to betray their partner in loss-framed and non-socially framed contexts. These effects did not interact with Dark Triad scores. Our data are consistent with the view that seemingly negative personality traits (like those associated with the Dark Triad traits) that persist in the population may serve as evolutionarily adaptive behavioral strategies.

## Introduction

When making decisions about scarce resources, what factors shape our behaviors? This paper focuses on understanding the interaction between personality traits and contextual factors in predicting selfish behavior. Specifically, we ask whether the presence of Dark Triad personality traits predict selfish behavior in a one-shot Prisoner’s Dilemma, a classic cooperation problem where the outcome of the game (and therefore the distribution of resources) is determined by players’ decisions to either cooperate with or betray their partner [1,2]. Because social and economic dilemmas do not occur in a vacuum, we also explore how framing a decision problem (e.g., in terms of a gain or loss; in social vs. nonsocial terms) impacts cooperative behavior and interacts with personality traits [3].

The Dark triad traits are a set of interrelated, but distinct, sub-clinical personality traits: Machiavellianism, narcissism, and psychopathy [4]. They are associated with a short-term and exploitative mating strategy, impulsivity, low self-control, risk-seeking behavior, future-discounting, aggression, and selfishness [5–9]. We were interested in understanding whether the Dark Triad personality traits might also be able to predict behavior—in this case, self-maximizing, uncooperative behavior—in these economic games. We arrived at this question after attempting to solve a puzzle: how is it that such apparently socially maladaptive traits (like those that typify the Dark Triad) could persist at a non-zero frequency in the human population? Like others [10], we suspected that despite appearing to be negative, Dark Triad personality traits might confer an advantage if they allow humans to maximize their access to resources by behaving selfishly, at least in certain contexts. There is substantial research which suggests that other personality traits (e.g., extraversion, agreeableness) predict cooperation in social and economic games [11–14]. Thus, we tested whether it was possible to predict self-maximizing behavior in a social and economic dilemma from Dark Triad personality score.

In order to understand how Dark Triad traits might support selfish behavior, it is important to first understand each component (psychopathy, narcissism, and Machiavellianism) of the Dark Triad separately. In its original description in the clinical literature, psychopathy was characterized as being comprised of a combination of impulsivity and antisocial tendencies, along with, anxiety, fearlessness, and a lack of remorse [15]. It is now generally thought that psychopathy consists of two factors, an interpersonal factor constituted by remorseless and the exploitative use of others, and an unstable, antisocial lifestyle factor constituted by impulsivity and poor behavioral control [16]. Narcissism generally captures a sense of superiority and impulsivity, grandiosity, dominance, and entitlement [17,18]. Machiavellianism is characterized by a cynical world view, lack of morality, and manipulativeness, especially for personal gain [19].

Machiavellianism might be especially likely to be related to performance in social dilemmas. Individuals high in Machiavellianism are characterized by their opportunistic and flexible behavior in social interactions [20–22]. Relatedly, Machiavellianism has been shown to be predictive of success in some social dilemmas. Gunnthorsdottir et al. [23] found that in a two-person one-shot constituent game in which participants could choose between reciprocation and defection, those high in Machiavellianism were much more likely to defect when it was in their advantage to do so. Bereczkei and Czibor [24] found that Machiavellianism was negatively correlated with contributions in a public good game and positively correlated with total profit gained by the end of the game. Other studies have found similar results, namely that individuals high in Machiavellianism contributed less to the public good and achieved relatively higher levels of personal gain in a public goods game [25,26]. These findings suggest that individuals high in Machiavellianism may be especially able to exploit others in contexts in which they stand to gain.

While the traits of the Dark Triad likely correspond to unique behavioral strategies, they are united by their common core of disagreeableness, manipulation, callousness, and low levels of the HEXACO Honesty-Humility factor [27,28,4]. It is this shared core of antisocial behavior that we were mainly interested in exploring in order to determine how differences in personality can relate to selfish behavior. However, in keeping with the substantial literature showing that Machiavellianism is strongly predictive of self-maximizing behavior in social dilemmas [20,23,24,25,26], we were also interested in whether Machiavellianism, narcissism, and psychopathy might uniquely predict selfish behavior or interact with the framing of the Prisoner’s Dilemma.

Of course, we recognized that economic and social decisions are not made in a vacuum, and the context surrounding such decisions plays a strong role. Thus, in addition to considering how personality traits may shape social and economic decisions, we also asked whether the framing of such decisions shaped performance. We know that the framing of a situation frequently impacts social and resource-allocational strategies: individuals are more likely to engage in self-maximizing behavior when the outcome of an economic game is framed as a loss relative to a gain, suggesting that humans are loss-averse [29–32]. Goerg, Rand, & Walkowitz [33] found that participants were more cooperative in a give frame than a take frame when completing a continuous version of the Prisoner’s Dilemma. Individuals are also less likely to harm others when an economic game uses non-neutral (e.g., social) language in task instructions [34–37], especially when decisions are framed as the morally right thing to do [38,39]. More generally, the extent to which a resource dilemma is framed as a social dilemma impacts rates of self-maximizing behavior, increasing cooperation [40]. These observations led us to ask whether framing (gain vs. loss; social vs. non-social) impacted performance in the Prisoner’s Dilemma (described below). We were interested both in the extent to which framing effects emerged in this task (which has rarely been tested; but see [41]), and also whether they interacted with Dark Triad personality traits.

In the present study, we measured participants’ level of Dark Triad (narcissism, Machiavellianism, and psychopathy), and empathy personality traits, and asked them to participate in a hypothetical, one-shot, version of the Prisoner’s Dilemma. The Prisoner’s Dilemma is a two-person social cooperation problem where the outcome of the game is determined by both player’s decisions to either cooperate or defect; the “rational” self-maximizing decision is to defect [1,2,42] (Axelrod, 1980; Axelrod, 1987; Rapoport & Chammah, 1965). While some describe the Prisoner’s Dilemma as an excellent test of cooperation [43,44], the one-shot version of this task also allows us to test self-maximizing behavior. This is because, in order to maximize one’s own outcome in the one-shot Prisoner’s Dilemma, one must defect (betray). Like the iterated version of the Prisoner’s Dilemma, the places social cooperation in conflict with personal gain. However, unlike the iterated version, the one-shot version minimizes the potential effects of revenge and reciprocity (since there is no opportunity to respond to another player’s choice). The choice of a one-shot Prisoner’s Dilemma therefore allows us to draw different conclusions than other work that has attempted to relate the Dark Triad traits to performance in an iterated Prisoner’s Dilemma task. For example, while Malesza [45] recently found a relationship between defection on the Prisoner’s Dilemma and the Dark Triad, because her task was iterated, we cannot know whether this relationship is due to selfish resource maximization, concerns related to revenge and reciprocity, or some combination of the two. If a relationship between the Dark Triad traits and Prisoner’s Dilemma performance emerges during a one-shot hypothetical Prisoner’s Dilemma, we can rule out revenge and reciprocity as necessary factors guiding this relationship.

In a preregistered 2 × 2 between-subjects design, we manipulated whether participants saw a gain- or loss- framed Prisoner’s Dilemma, and whether the Prisoner’s Dilemma was described as a social or non-social game. If loss framing affects behavior by making people risk averse, then participants should defect more in the loss than the gain framed Prisoner’s Dilemma. Additionally, if framing a dilemma socially increases cooperation, then we expect less defection in the social compared to the non-social Prisoner’s Dilemma. We also related performance on the Prisoner’s Dilemma to the extent to which participants displayed Dark Triad personality traits. If it is the case that the Dark Triad traits predispose individuals to engage in self-maximizing behavior, then the Dark Triad should predict defection in the Prisoner’s Dilemma.

## Method

### Participants

We tested 1798 participants, aged 18–74 years (*M* = 32.2). Participants received US$2 on Amazon’s Mechanical Turk crowd-sourcing website; we used TurkPrime to recruit [46,47]. Data collected online using Mechanical Turk has been found to be comparable to data collected in the lab [48,49]. Unlike previous work on this topic (e.g., [45]), we aimed to recruit a very large and diverse sample of participants, in order to increase the generalizability of our work: Participants (1) were located in either the US or India and had (2) completed between 100 and 10,000 HITs; (3) a HIT Approval rate between 79–100%, and (4) a unique IP address. Based on previous research demonstrating cross-cultural variability in performance on economic games and on scores of personality measures [50–53], we recruited participants from both the United States and India in order to have a high-variability and high-generalizability dataset.

### Procedure

Participants provided consent, and were told that they would (1) play a quick online game with another participant and then (2) complete some personality measures. Participants were randomly assigned to one of four conditions. We had a 2 (Social vs. Non-Social Condition) × 2 (Gain vs. Loss Framing) between-subjects design.

Participants read a paragraph introducing them to the Prisoner’s Dilemma [2], and its accompanying payoff matrix. Importantly, rewards in our Prisoner’s Dilemma were hypothetical and not incentivized with actual monetary rewards. Past work suggests that whether rewards are real or hypothetical has little impact on cooperative behavior in social dilemmas [54,55], (but see [56]; for a review see [57]). Because our primary research questions focused on detecting relationships between personality traits and PD performance (and not on base rates of defection), we did not anticipate that using a hypothetical PD would impact our main analyses.

The content of the Prisoner’s Dilemma (PD) and accompanying matrix differed by condition. Specifically, the PD in the Social condition was framed so that participants could “Cooperate” or “Betray” a “friend”, and the consequences of the Prisoner’s Dilemma were described in terms of years of freedom and time with friends/family gained/lost. In the Non-Social condition, the PD was framed as a game played with a stranger (referred to as the “other player”), and with neutral decision choices (“Option A/B” rather than “Cooperate/Betray”); the consequences were described in terms of points gained/lost (see S1 File for full script and materials).

We also manipulated whether the Prisoner’s Dilemma was described using a Gain or a Loss Frame. For the Gain frame, the consequences of the Prisoner’s Dilemma were explained in terms points (non-social) or years of freedom (social) gained (e.g., “If you both choose Option A, you will each earn 10 points”). For the Loss frame, the Prisoner’s Dilemma was explained in terms of points/years in jail that result in time lost with friends and family (e.g., “If you both choose Option A, you will each lose 10 points”). Consistent with this, the payoff matrix depicted how many points/years in jail participants would gain/lose depending on the condition.

Because previous research (e.g., [58]) has shown that socially-framed tasks are easiest to understand, we wanted to ensure that none of our effects could be attributed to a failure to understand the consequences of the actions in the Prisoner’s Dilemma. Just as previous researchers have sometimes checked for comprehension of the payoff matrix [45,59,60], we required participants to answer four comprehension questions about the payoff matrix prior to completing the Prisoner’s Dilemma. Specifically, participants were asked about their expected payoff outcome for every possible outcome (e.g. “If you and Player 1 both [choose to cooperate/betray each other]/[choose option A/option B], how many [years free/points will you gain/lose]?”) and were presented with four possible answers (three or four of the values within their payoff matrix). Participants were alerted if they answered a question incorrectly and could not continue to the rest of the experiment until they successfully identified the outcomes for every contingency (they Cooperate vs. Defect * their partner Cooperates vs. Defects).

After the comprehension questions, participants proceeded to a screen with a loading symbol, and were told that they were being connected to another player online. Participants were not actually connected to another player but were led to believe so in order to make their behavioral responses as realistic as possible (i.e. not playing against a computer). After the connection was “successful” (after about six seconds), participants were presented with the Prisoner’s Dilemma matrix again and asked to make their decision to either [“Cooperate” or “Betray”] in the Social condition or [choose “Option A” or “Option B”] in the Nonsocial Condition. After making their decision, participants were “disconnected” from their partner and proceeded to a screen introducing the second part of the study, the personality measures (described below).

During the personality measures portion, participants were instructed to answer the questions as accurately as possible and were told that they could skip any questions that made them uncomfortable. The Dark Triad traits were assessed using the Short Dark Triad [61], a 27-item self-report questionnaire consisting of three subscales: psychopathy (e.g., “Payback needs to be quick and nasty”), Machiavellianism (e.g., “It’s not wise to tell your secrets”), and narcissism (e.g., “I have been compared to famous people”). Participants also completed the Basic Empathy Scale [62], a 20-item self-report questionnaire which measures Cognitive (e.g., “I can usually work out when people are cheerful.”) and Affective empathy (e.g., “I get caught up in other people’s feelings easily”). Within the survey, there were two attention checks, one placed within the Short Dark Triad and the other in the Basic Empathy Scale: “If you’re paying attention select somewhat disagree” and “If you’re paying attention select strongly agree”.

Next, participants completed the Life History scale, an in-house scale that consisted of six questions in total, three measuring reproductive strategy and three measuring perceptions of death (e.g., “How many siblings do you have?”, “At what age do you think you will die?”). We created our own simplified Life History Scale because we were concerned about the cross-cultural generalizability of several existing scales (e.g., with respect to questions about long-term mate choice etc…; see S1 File for more information). Participants then answered demographic questions about their age, race, country of origin (USA, India, or other), and childhood socioeconomic status. Upon completion of the survey, participants were thanked and debriefed.

## Results

### Exclusions

Following plans outlined in our preregistration (https://osf.io/knm7u/), of our 1798 participants, we did not include the 194 participants who failed to complete the survey in its entirety, allowing us to exceed our target of 1600 participants to arrive at an *n* of 1604.

As planned, we next excluded participants who failed one or more of the attention checks (i.e. “If you’re paying attention select somewhat disagree” and “If you’re paying attention select strongly agree”) within the personality measures (*n* = 204). This led to a final *n* of 1400 for our analyses. We also excluded some individual responses at the question level (detailed below) prior to any additional transformations or analyses. For these exclusions, only the relevant outlier responses were excluded; all other data remained in the dataset. We excluded responses to the life history questions that were absurd or failed to answer the question (e.g., responses of 2060, 1, or 0 to the question “At what age do you think you will die?”; *n* = 233). We had not anticipated these outlier responses in our preregistration, and as such, these exclusions represent a deviation from our preregistered plan. Still, we believe that responses of 0 and 2060 do not likely reveal participants’ true beliefs about their anticipated age at death (see S1 File for full exclusion criteria). We also asked participants in the survey to indicate their country of origin (US, India, or other). For 333 participants, we could not determine their country of origin with certainty (either because they failed to respond to the question or because their response conflicted with information provided by Mechanical Turk)—for analyses that included country as a variable we only used the data for which we could confidently determine country of origin (*n* = 1067).

### Data management and scale construction

Data were cleaned and compiled according to guidelines outlined in our preregistration (https://osf.io/knm7u/); we have redundantly detailed our protocols in the S1 File as well.

#### The Dark Triad score

Because some previous research has treated the Dark Triad traits as a composite reflecting a single, underlying social strategy [6], and as we had no *a priori* hypothesis regarding the Dark Triad subscales, we preregistered our analyses with the intention of treating the Dark Triad traits as a composite (by taking the mean DT score across all items). Other work on the Dark Triad traits has used Principal Components Analysis (PCA) to capture the shared variance across items on the scale [63]. To ensure that our analytic choice to create a DT composite was appropriate, we also conducted a post-hoc Principal Components Analysis (PCA) on the Dark Triad scale, which allowed us to capture the shared variance across the instrument. The first component (PC1) was correlated with our Dark Triad composite score with an *r*^2^ > .99 if the PCA was conducted on the three subscale subscores, and an *r*^2^ > .97 if the PCA was conducted on all 27 items in the SD3. This strongly suggests that our preregistered Dark Triad compositing process (taking the average) was appropriately similar to other ways of calculating Dark Triad composites (e.g., via PCA; see S1 File). Importantly, the reliability for our composite Dark Triad score was comparable to (alpha = .74 when calculated over the three subscale averages) or higher than (alpha = .88 when calculated over all 27 individual items) each individual scale (alpha = .82_Machiavellianism_; .78_Narcisism_; .76_Psychopathy_).

At the outset, in addition to our continuous measure of Dark Triad, we also planned to treat our Dark Triad measure as a categorical variable—in particular, we planned to compare performance on those who had scores in the top quartile to those who had scores in the bottom quartile. We made this choice because, while categorical variables certainly lose information, it allowed us to make claims about the relative performance of those who were high vs. low in Dark Triad. This is important because, at the outset, we believed it was possible that only those individuals with very high levels of the Dark Triad would demonstrate self-maximizing behavior. For this reason, we coded participants who were in the top and bottom quartiles for Dark Triad scores as being High and Low in Dark Triad, respectively.

As described above, our preregistered intention was to treat the Dark Triad traits as a composite, as we had no *a priori* hypotheses about the relationship between particular subscales of the SD3 and Prisoner’s Dilemma performance. Instead, we planned from the outset to report subscale-specific analyses as exploratory, and to place them in supplemental materials. However, in keeping with research that has predicted performance from a model containing all three subscale scores [64–67], some of the literature on the unique role of Machiavellianism in shaping behavior (e.g., [24]), and comments from colleagues, we reconsidered our initial analytic choice post-hoc. Thus, in addition to our planned analyses of the Dark Triad composite, we also report exploratory models in which we predict performance from Machiavellianism, narcissism, and psychopathy. This also allowed us to more explicitly test hypotheses about the potentially unique relationship between Machiavellianism and Prisoner’s Dilemma performance.

### Analyses

#### Preliminary analyses

Correlations for each measure in our study are presented in Table 1.

**Table 1:** Descriptives and Pearson’s correlation coefficients between Dark Triad, empathy, and life history.

	*M*	*SD*	1	2	3	4	5	6	7	8
1. Dark Triad	2.83	0.59	-	.84**	.77**	.84**	-.30**	-.23**	-.31**	.11**
2. Mach	3.33	0.75		-	.43**	.61**	-.24**	-.18**	-.25**	.08**
3. Narc	2.82	0.73			-	.46**	-.14**	-.12**	-.12**	.06*
4. Psych	2.33	0.71				-	-.37**	-.27**	-.39**	.14**
5. Empathy	61.51	8.48					-	.91**	.81**	-.02
6. Aff Emp	32.54	5.7						-	.49**	-.08*
7. Cogn Emp	28.97	4.04							-	.02
8. Life History	-0.02	0.48								-

* *p* < .05; two-tailed.

** *p* < .01; two-tailed.

#### Dark Triad and Prisoner’s Dilemma

**Fig 1:**
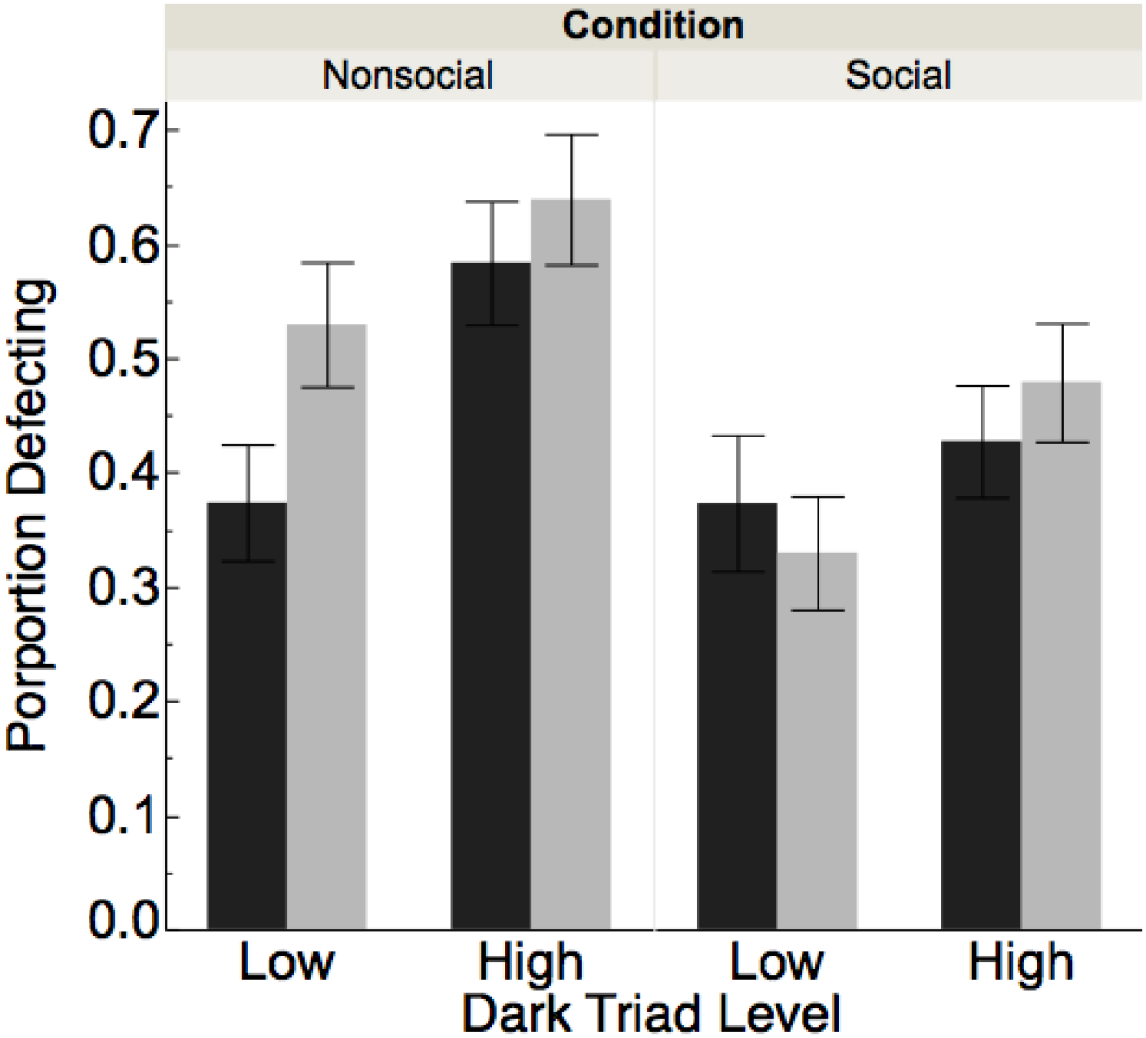
Proportion defecting for those high vs. low in Dark Triad, by condition (Social, left vs. non-social, right) and framing (loss, gray vs. gain, black).

To determine the role of the Dark Triad traits in behavior in the Prisoner’s dilemma, we first constructed models predicting Defection (a binary variable) from Dark Triad score [68]. For this and all models with continuous variables as predictors, we scaled our continuous variables in order to facilitate interpretation. We found that the Dark Triad score predicted Defection in the model (*B* = 0.16, *SE* = 0.05, *p* = .002, OR: 1.18) with the 95% confidence interval showing odds ratios just over 1 (CI = 1.06, 1.31). As planned in our preregistration, we also compared defection rates for those who were High vs. Low in the Dark Triad on the logic that there might be unique differences in behavior at the extreme ends of the Dark Triad. We found that those high in the Dark Triad were more likely to defect (52.1% defection) than those low (40.1% defection; χ^2^(1) = 9.95, *p* = .002). See Fig 1 for a visualization of cooperation by Dark Triad level.

#### Dark Triad, framing, condition and Prisoner’s Dilemma

We next tested whether framing (Gain vs. Loss) affected behavior in the Prisoner’s Dilemma. We found that participants were more likely to Defect in the Loss (Defection: 49.3%) than the Gain condition (42.1% Defection; χ^2^(1) = 7.19, *p* = .007). We then constructed a model predicting Defection from Dark Triad (standardized continuous), Framing (Gain: .5, Loss: -.5; note that for framing and condition, the decision to center was post-hoc), and their interaction. We found that Framing (*B* = -.29, *SE* = 0.11, *p* = .007, OR: 0.75, CI: 0.60, 0.92) and Dark Triad score (*B* = .17, *SE* = .05, *p* = .002, OR: 1.18, CI: 1.06, 1.32) were both significant predictors of Defection, and there was no interaction (*B* = -.004, *SE* = 0.11, *p* = .97).

We next asked whether participants behaved differently in the Social vs. Non-Social Conditions of the Prisoner’s Dilemmas. We found that participants defected less often in the Social Condition (Defection: 36.2%) relative to the Non-Social Condition (Defection: 55.3%; χ^2^(1) = 51.55, *p* < .001). We then tested the relation between Dark Triad score (standardized continuous) and Social Condition (Social: .5, Non-Social: -.5) in predicting Defection. If it is the case that Dark Triad traits allows people to maximize their gains (regardless of the social consequences), then participants high in Dark Triad traits should have high rates of defection in both the Social and Non-Social conditions. We found an effect of Social Condition (*B* = -0.81, *SE* = 0.11, *p* < .001, OR: 0.45, CI: 0.36, 0.55) and Dark Triad (*B* = 0.19, *SE* = .06, *p* < .001, OR: 1.21, CI: 1.09, 1.36), but no interaction (*B* = -0.16, *SE* = 0.11, *p* = .159).

#### Subscales, framing, condition and Prisoner’s Dilemma

At the outset we had intended to analyze only our Dark Triad composite and categorical classification. However, as described above, we made the post-hoc decision to conduct the above analyses again, but this time predicting performance from all three subscales. We had initially been concerned about issues of collinearity in adopting this approach. However, the variance inflation factor (VIF) was below 3, indicating that while the factors were correlated, collinearity was not a concern in this case.

**Fig 2:**
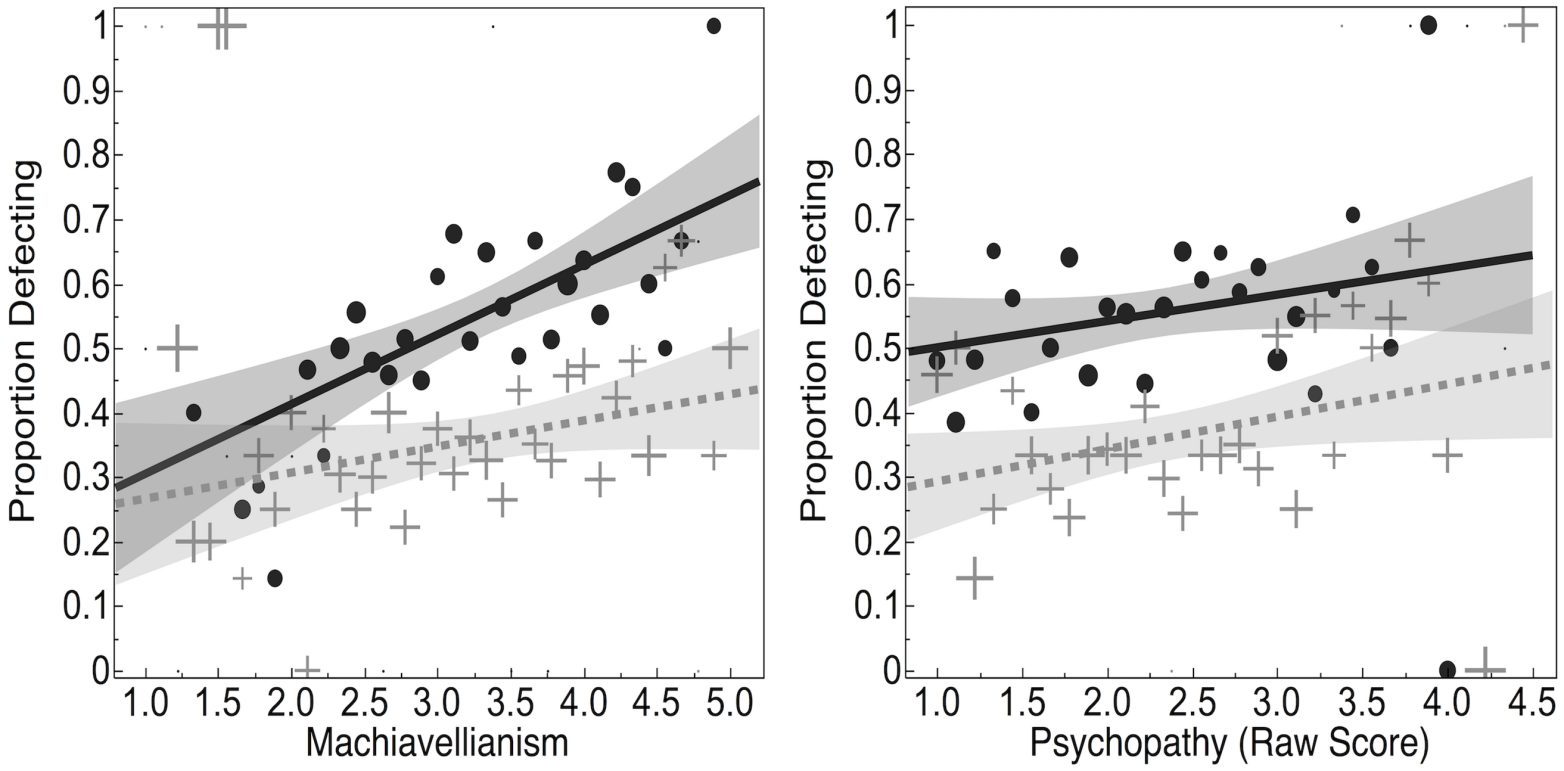
Proportion defecting, in relation to Machiavellianism (left) and psychopathy (right), by social (gray, dotted, cross) and non-social (black, solid, circle) conditions. Marker size increases as sample size increases.

When predicting defection from psychopathy, Machiavellianism, and narcissism, we found that only Machiavellianism was a significant predictor (*B* = .211, *SE* = .069, *p* = .002, OR: 1.24, CI: 1.08, 1.42), while psychopathy (*B* = -.022, *SE* = .069, *p* = .753, OR: 0.98, CI: 0.85, 1.12) and narcissism were not (*B* = .012, *SE* = .062, *p* = .853, OR: 1.01, CI: 0.89, 1.14). We then constructed a model predicting Defection from psychopathy, narcissism, Machiavellianism, Framing, Condition, and their interactions. We again found that Machiavellianism (*B* = .208, *SE* = .072, *p* = .004, OR: 1.23, CI: 1.07, 1.42), Condition (*B* = -.812, *SE* = .111, *p* < .001, OR: 0.44, CI: 0.36, 0.55), and Framing (*B* = -.288, *SE* = .111, *p* = .01, OR: 0.75, CI: 0.60, 0.93) were significant predictors of Defection while narcissism (*B* = -.008, *SE* = .065, *p* = .898, OR: 0.99, CI: 0.87, 1.13) and psychopathy (*B* = .023, *SE* = .073, *p* = .753, OR: 1.02, CI: 0.89, 1.18) were not. We also found significant interactions between Machiavellianism and Condition (*B* = -.29, *SE* = .144, *p* = .044, OR: 0.75, CI: 0.56, 0.99) and psychopathy and Condition (*B* = .308, *SE* = .146, *p* = .034, OR: 1.36, CI: 1.02, 1.81), such that increases in Machiavellianism predicted smaller increases in defection rates in the Social Condition than in the Non-Social condition. See Fig 2 for a visualization of these interactions. No other interactions reached our significance threshold (*p* > .099).

#### Other analyses

As planned, we also asked whether the Life History measure and Empathy predicted defection in the Prisoner’s Dilemma. Neither predicted defection in the Prisoner’s Dilemma, or interacted with framing (see S1 File for full reporting of these analyses). We also conducted analyses testing the effect of country on performance; while we found effects of country and these are presented in the S1 File, we were unable to assess whether our measures were structurally invariant, and thus these data should be interpreted with caution. In addition, we conducted post-hoc analyses of the impact of participant sex on performance, and found that it did not have an impact (see S1 File).

## Discussion

We asked whether personality traits and framing interact to encourage self-maximizing behavior in social and economic dilemmas. To test this, participants played a hypothetical version of the one-shot Prisoner’s Dilemma online, but saw the task framed as either a social or non-social task, and in terms of potential gain or potential loss. We also measured participants’ Dark Triad traits. We had two main findings. First, we found that the Dark Triad traits predicted defection in the Prisoner’s dilemma, and that this effect was most evident for Machiavellianism. Second, we found that participants were significantly more likely to defect in the loss and the non-social conditions compared to the gain and socially framed Prisoner’s Dilemmas, respectively. Together, this suggests that Dark Triad personality traits—and especially Machiavellianism—underlie self-maximizing behavior in a social/economic dilemma. Our findings also suggest that and that framing a resource dilemma in terms of a gain or socially makes people more likely to behave cooperatively.

At the outset, we had wondered whether Dark Triad personality traits might interact with framing; previous research had found such a relationship between personality and gain-loss framing such that “prosocial” were more likely to cooperate in loss than gain frames and “competitors” cooperated very little in both loss and gain framed dilemmas [41]. We predicted that if the Dark Triad traits confer an advantage because they lead to self-maximizing behavior, then participants high in Dark Triad traits would be likely to defect in both the loss and gain frame. Conversely, we predicted that if the Dark Triad traits offers an advantage because it confers an individualist mindset (i.e., that the participant wants to maximize their own outcomes without harming another’s), then they should defect especially often in a loss, relative to gain. We did not find any interactions between the Dark Triad composite and framing of the Prisoner’s Dilemma. This is consistent with the view that, overall, the Dark Triad traits confer an advantage because they lead to self-maximizing behavior regardless of context.

Why might there be a relation between the Dark Triad traits and self-maximizing behavior in the Prisoner’s Dilemma? Researchers who adopt an evolutionary approach to understanding individual differences have suggested that the Dark Triad traits might underlie an exploitative interpersonal social strategy [5,8]. In this evolutionary framework, personality traits can be viewed as adaptive “social strategies” (i.e., unconscious and automatic behavioral response patterns), that confer specific fitness benefits depending on the context [69]. In other words, those who possess a particular set of personality traits gain an adaptive advantage over others in certain contexts, and this allows them to pass on their genes at a rate they couldn’t otherwise. Our results, that Dark Triad score predicted defection in the Prisoner’s Dilemma, are what we would expect if it were the case that Dark Triad traits constitute an evolved behavioral strategy that emphasizes self-interest. Our results are also consistent with the possibility that Machiavellianism, in particular, allows individuals to maximize self-interest.

Our finding, while exploratory, that Machiavellianism was a unique predictor of defection is consistent with past work which has found that Machiavellianism relates to exploitation in social dilemmas [20,23,24,25,26,45]. Our findings provide support for the notion that Machiavellian people are especially likely to successfully exploit others, and to gain from doing so [70]. Our work builds on Malesza’s [45] finding that Machiavellianism predicts defection in an iterated Prisoner’s Dilemma; because we found the same relationship in a one-shot prisoner’s dilemma (where there is no opportunity for revenge and no expectation of reciprocity), our data suggest that Machiavellianism predicts Prisoner’s Dilemma performance because it encourages self-maximizing behavior, and not (only) because it may predispose individuals to take revenge or have low expectations of reciprocity.

Our finding that Machiavellianism predicts defection in the Prisoner’s Dilemma echoes previous research on this topic in another way, too. Past research has suggested that individuals high in Machiavellianism are sensitive to social context. For example, they contribute more in a public goods game (in which their own contributions would be known to others) when there are larger numbers of altruists in their group [24]. Our results support this idea that Machiavellians are especially sensitive to the context of the social dilemma, and that this may cause them to avoid exploiting others in social contexts in which reciprocal relationships (e.g., friendships) are invoked. In particular, we found that while the likelihood of defection increased substantially with Machiavellianism score in the non-social condition, it increased less substantially in the non-social condition. In other words, individuals high in Machiavellianism were somewhat less likely to defect in the social condition, relative to the non-social condition. While this is consistent with previous work, we wish to encourage caution in interpreting this finding for two reasons. First, analyses involving Machiavellianism were post-hoc and exploratory. Second, neither our post-hoc exploratory nor confirmatory factor analyses (see S1 File) provided evidence that Machiavellianism was a separate factor in the SD3 from narcissism and psychopathy, and so our analyses involving Machiavellianism may be limited by our ability to use the SD3 to accurately assess Machiavellianism.

Our results also suggest that the Dark Triad might be associated with a lack of morality. In a study exploring how labeling options in an economic game as moral, [38] found that people show a general morality preference. That is, prosocial people tend to select the option that is framed as morally right. Our findings suggest that those individuals who are high in the Dark Triad, specifically Machiavellianism, demonstrate a disregard for what is framed as morally right. Future research should explore whether those high in the Dark Triad lack this morality preference.

One strength of the present study was its use of a large sample, and a sample drawn from more than one country [71]. In order to increase the variability within our data and the generalizability of our conclusions, we decided at the outset to collect data from India and the US. We selected India because previous research had found that participants from India are more punitive than their US counterparts in economic games [50], and that Indian participants scored higher in the Dark Triad traits than US participants [51]. Additionally, other work has found that participants from India are sometimes less cooperative than their American counterparts [72]. Our intention in including two nationalities in our dataset was not to do specifically comparative work, nor to encourage reductionist conclusions about social processes in particular nations; instead, we hope that future researchers will continue to study social processes across diverse and large samples. While we report differences in performance based on nationality in our S1 File, because we were unable to test for the structural invariance of the SD3 in our US vs. Indian populations, we encourage readers to be somewhat skeptical of these analyses.

There are several limitations to our present study that require us to exercise caution in interpreting some of our findings. First, because we sampled participants online, we were not able to ensure an equal distribution of men and women within each sample; in our Indian dataset, for example, there were more than twice as many men as women. For this reason, it is difficult to draw strong conclusions about the impact of demographics on performance on our task. Second, while we believe our design and manipulations were effectively understood by our participants, it is important to note that our Social vs. Non-social framing manipulation manipulated more than *just* the social component—specifically, in the Social condition, the Prisoner’s Dilemma was framed with respect to a “friend”. Thus, this condition may have invoked not only social reasoning, but expectations of reciprocity. Future researchers interested in the effect of social vs. non-social framing on the Prisoner’s Dilemma might consider using social actors for whom no expectation of reciprocity exists (e.g., a stranger). A third limitation was our choice of Dark Triad scale (the SD3); while we were able to effectively use it as a composite, post-hoc Confirmatory Factor Analyses failed to indicate that the SD3 is composed of 3 unique subscales; and post-hoc Exploratory Factor Analyses failed to identify factors that could map on to the 3 unique subscales claimed to be tapped by the SD3 (see S1 File). Future research should further explore the psychometric properties of this measures.

Future research is needed to determine if the Dark Triad traits predict self-maximizing behavior across a range of social and economic contexts. More generally, future research will benefit from understanding the effects of culture in shaping (a) personality and (b) individuals’ approaches to social and economic dilemmas. While the current study found tentative evidence for cross-cultural difference in Dark Triad levels, the mechanisms guiding such differences are still unknown—we encourage researchers to explore our dataset in order to generate new exploratory hypotheses about the sources and consequence of personality, cultural, and contextual factors shaping social and economic reasoning.

## Supporting information

S1 File Supplementary materials. (DOCX)
