# Supplementary material for: The Dark Triad and framing effects predict selfish behavior in a one-shot Prisoner’s Dilemma
Source: PLoS One. 2018 Sep 19;13(9):e0203891. doi: 10.1371/journal.pone.0203891 (PMC6145542; doi:10.1371/journal.pone.0203891)
Supplement: S1 File — (DOCX) [file pone.0203891.s001.docx]

**Supplementary Online Materials**

Table of Contents

1. Prisoner’s Dilemma and Payoff Matrices……………………………………………....2
   1. Neutral Loss………………………………………………………………….....2
   2. Neutral Gain………………………………………………………………….....5
   3. Social Loss……………………………………………………………………...8
   4. Social Gain…………………………………………………………………….11
2. Additional Scales……………………………………………………………………...14
   1. Empathy……………………………………………………………………….14
   2. Life History Scale……………………………………………………………..14
   3. Childhood Socioeconomic status……………………………………………...14
3. Supplementary Analysis Plan Information……………………………………………15
   1. Exclusions for Life History Scale……………………………………………..15
   2. Score Compilation……………………………………………………………..15
4. Supplementary Results………………………………………………………………...16
   1. Dark Triad Subscale Analyses………………………………………………....16
   2. Multi-Group Confirmatory Factor Analysis…………………………………...19
   3. Life History…………………………………………………………………….20
   4. Empathy………………………………………………………………………..20

Exploratory Analyses…………………………………………………………………..21

- 1. Sex……………………………………………………………………………...21
  2. Country, Condition, Framing, Sex, and Dark Triad……………………………23
  3. References………………………………………………………………….…..25

**Prisoner’s Dilemma and Payoff Matrices**

**Neutral Loss Condition.**

***PD introduction.***

**
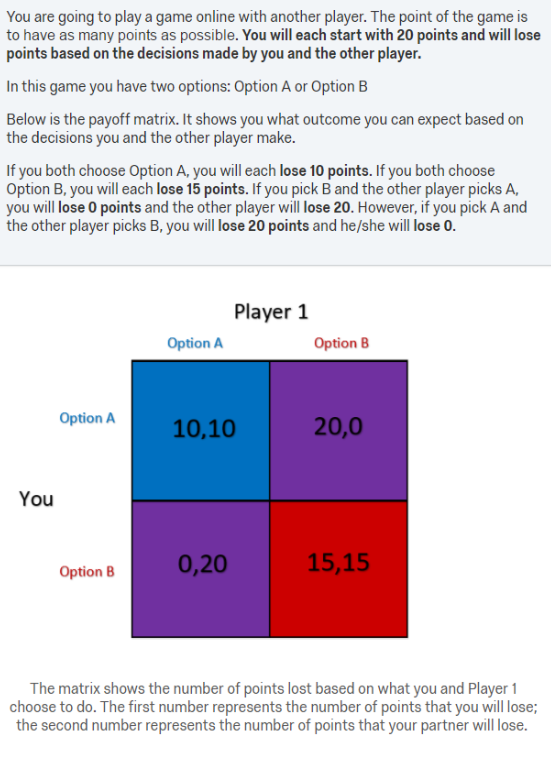
**

***Comprehension check.***

*
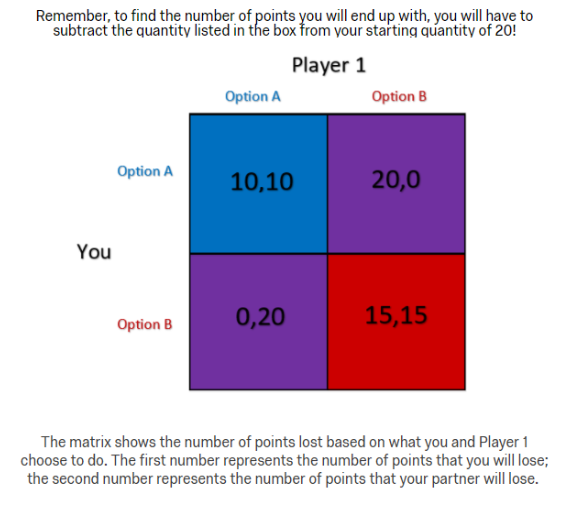

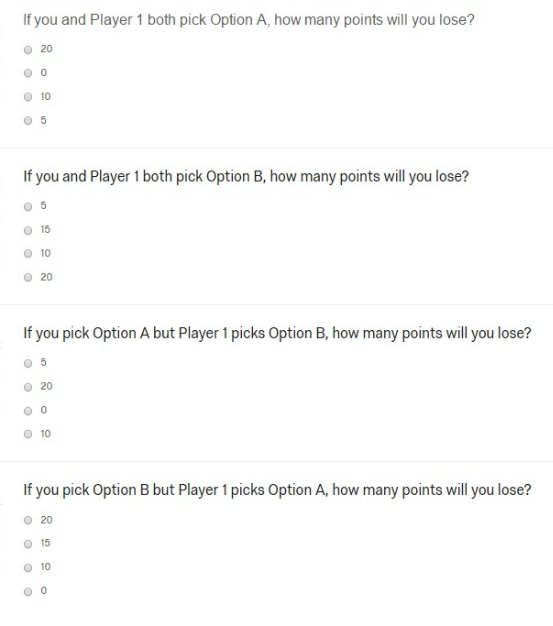
*

***Decision block.***

*
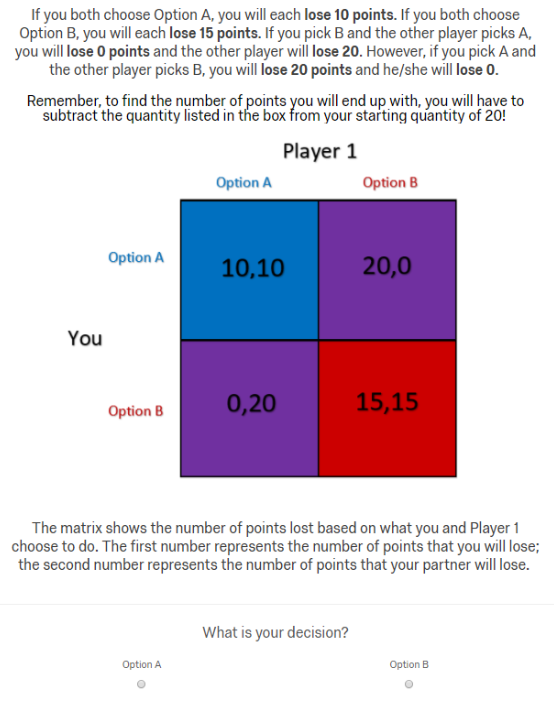
*

**Neutral Gain Condition.**

***PD introduction.***

*
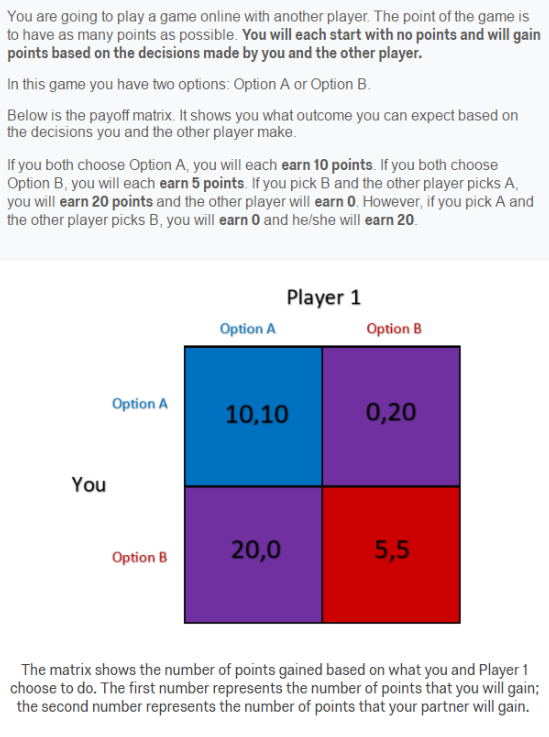
*

***Comprehension check.***

***
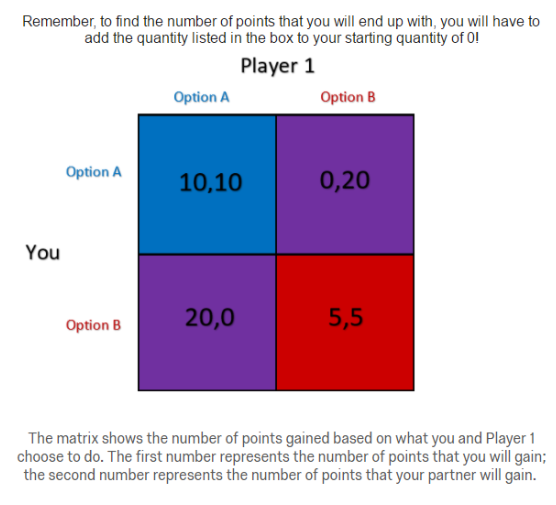
***

***
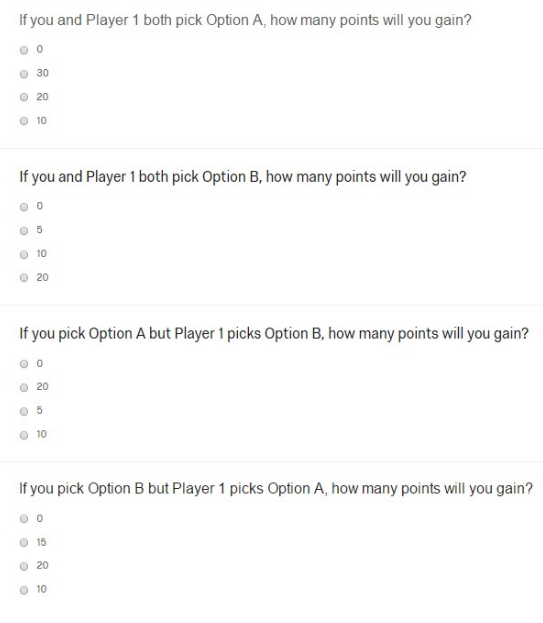
***

***Decision block.***

*
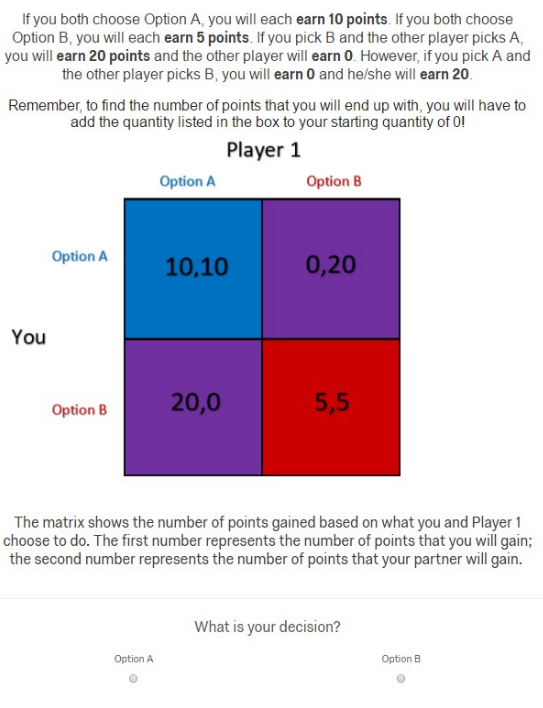
*

**Social Loss Condition.**

***PD introduction.***

***
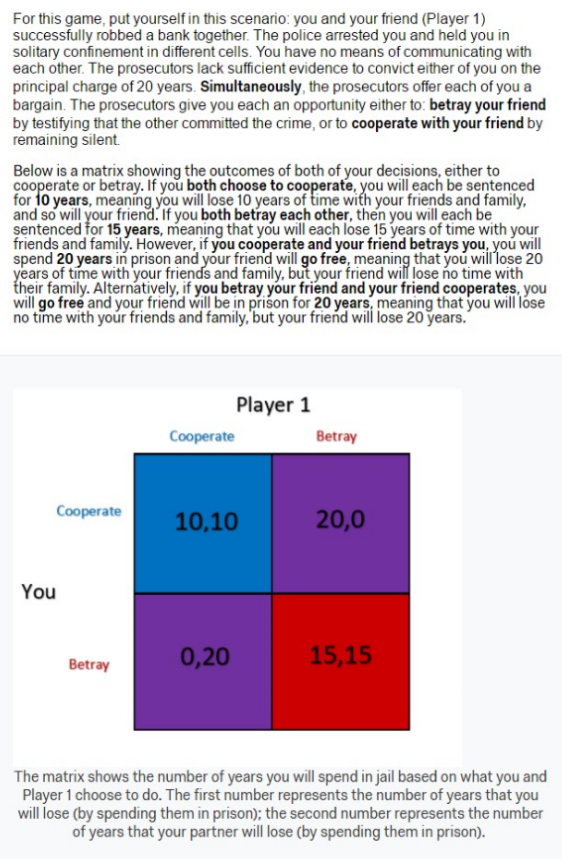
***

***Comprehension check.***

***
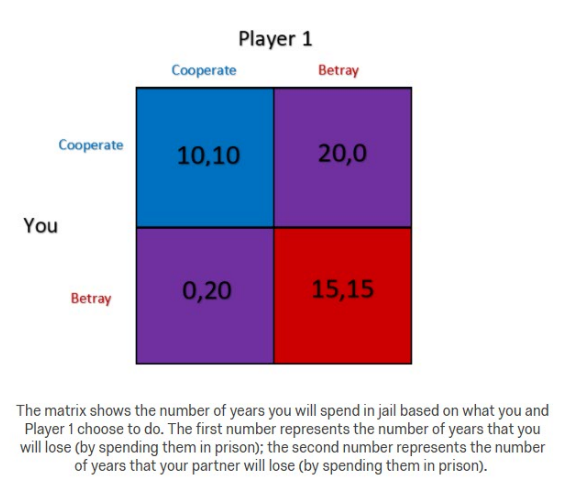
***

***
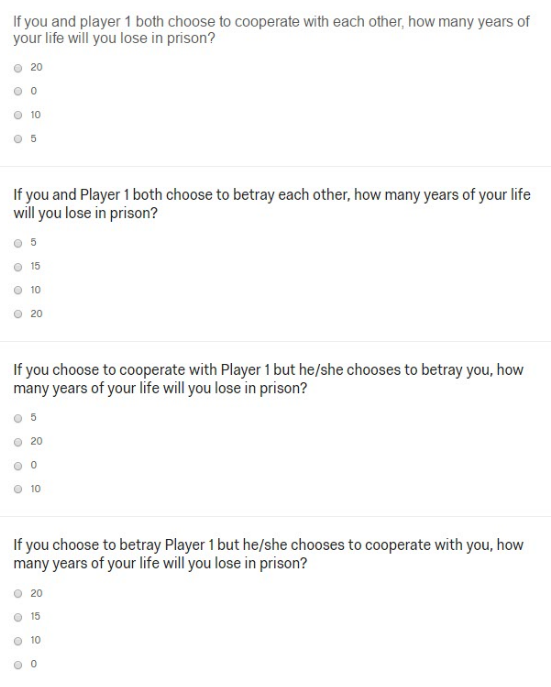
***

***Decision block.***

*
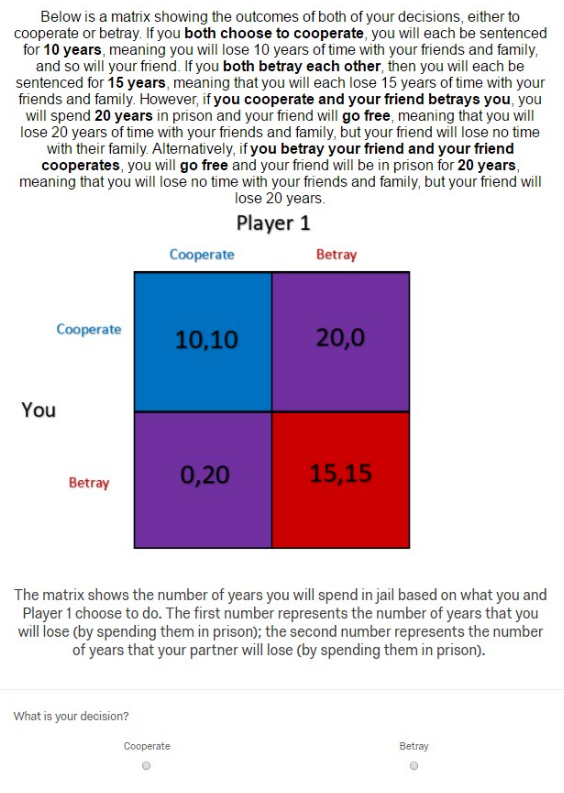
*

**Social Gain Condition.**

***PD introduction.***

***
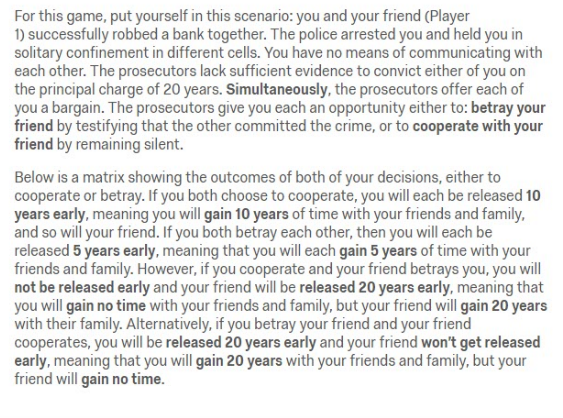
***

***
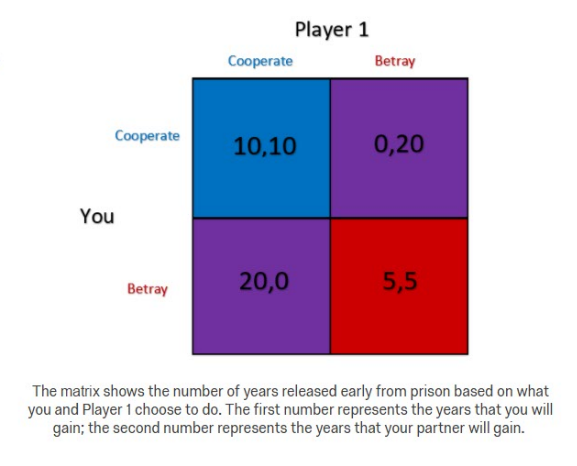
***

***Comprehension check.***

***
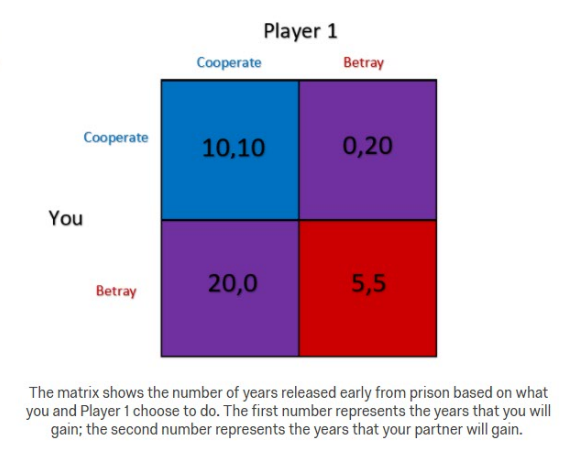
***

***
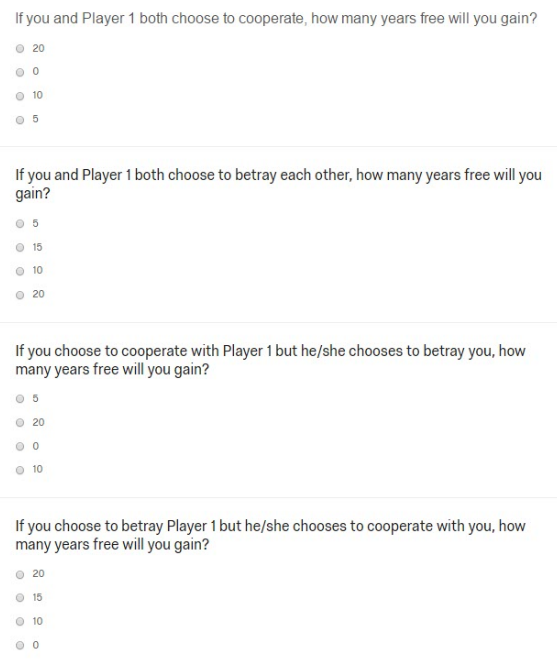
***

***Decision block.***

***
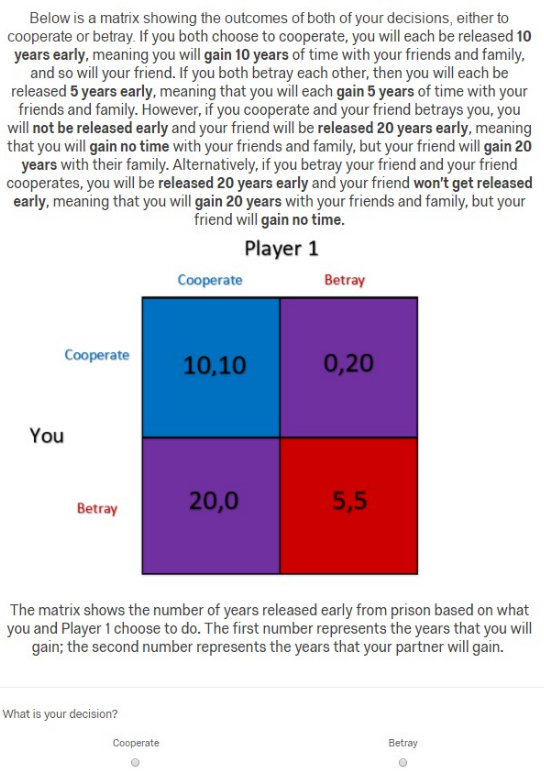
***

**Additional Scales**

**Empathy.** Participants typically indicate their agreement to empathy questions using a 5-point likert scale (1-*strongly disagree* to 5-*strongly agree*) but due to a computer error, the measure was on a four-point scale (1-*strongly disagree*, 2-*disagree*, 3-*agree*, 4-*strongly agree*). Responses were summed such that the scores for each question were added together to find the Affective (11 items) and Cognitive Empathy (9 items) measures. These measures were added to together to find the Global Empathy score.

**Life history scale*.*** We introduced this scale on the logic that the Dark Triad might be related to a fast life history strategy (Jonason, Koenig, & Tost, 2010) and as such might predict selfish behavior in the Prisoner’s Dilemma. Many of the best-normed Life History scales have been normed primarily in Western populations, and include items (e.g., about ease of securing a romantic date; proximity to nature) that we anticipated could be impacted by cultural factors in ways that could be, at minimum, complex (e.g., Copping, Campbell, & Muncer, 2014). This, we designed our own Life History measure.

The questions measuring Life History reproduction were all open response and included “How many children do you have or would like to have?”, “How many siblings do you have?”, and “At what age did you have your first child or at what age would you ideally want to have your first child?” The questions measuring Life History death perceptions included “At what age do you think you will die?”, “To what extent do you agree with the statement ‘people in my community die young’”, and “To what extent do you agree with the statement ‘on a day-to-day basis, I feel safe’”.

**Childhood socioeconomic status.** We also had three questions measuring childhood socioeconomic status which asked participants to rate their agreement using a 7-point likert scale (1-strongly disagree to 7-strongly agree). The questions included “My family usually had enough money for things when I was growing up”, “I grew up in a relatively wealthy neighborhood”, and “I felt relatively wealthy compared to the other kids in my school.” However, our intention was not to analyze these data (see preregistration here: https://osf.io/knm7u/), but instead to collect these data for potential exploratory analyses.

**Supplementary Analysis Plan Information**

**Exclusions for life history scale*.*** Responses were excluded for: “At what age do you think you will die” if they were >150 or <1; “How many children do you have or would like to have?” if they were >15; “How many siblings do you have?” if they were >30; and “At what age did you have your first child or at what age would you ideally want to have your first child?” if they were between 0-10 or >80.

**Score compilation*.*** Dark Triad scores were computed for each participant by averaging their scores on the three subsections for narcissism, psychopathy, and Machiavellianism to get a composite Dark Triad score. The Empathy score was found by summing responses to the 9 Cognitive and 11 Affective measures of Basic Empathy Scale. We created Life history scores by first z-scoring responses to the 6 Life History questions, then flipping the signs on questions 1 (“At what age do you think you will die?”), 3 (“To what extent do you agree with the statement ‘on a day-to-day basis, I feel safe’”), and 6 (“At what age did you have your first child or at what age would you ideally want to have your first child?”), and then finding the average z-score for each participant.

**Supplementary Results**


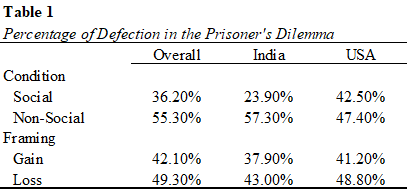


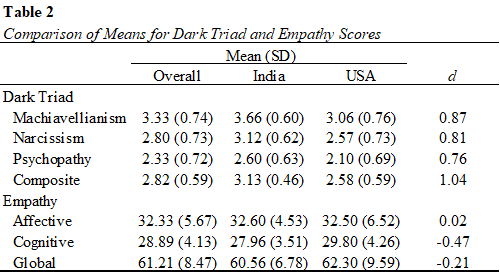


**Dark Triad Subscale Analyses.**

*Principal Components Analysis.* From the outset we planned on analyzing the SD3 composite measure as our main DV because we lacked *a priori* hypotheses about the relationship between Defection and each sub-trait, but rather hypothesized about the impact of being, on average, high on all three sub-traits. To assess concerns regarding this strategy, we conducted a post-hoc PCA (reported in main text) which suggested that our Dark Triad average measure picked up on the shared variance across items quite well. Note that the correlation between the first component and our Dark Triad score is remarkably high.


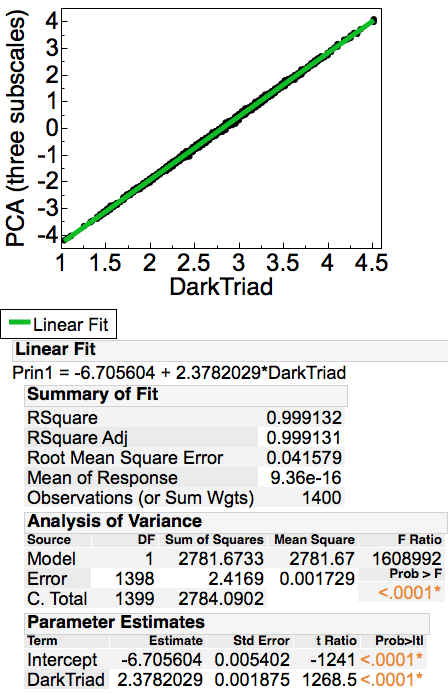

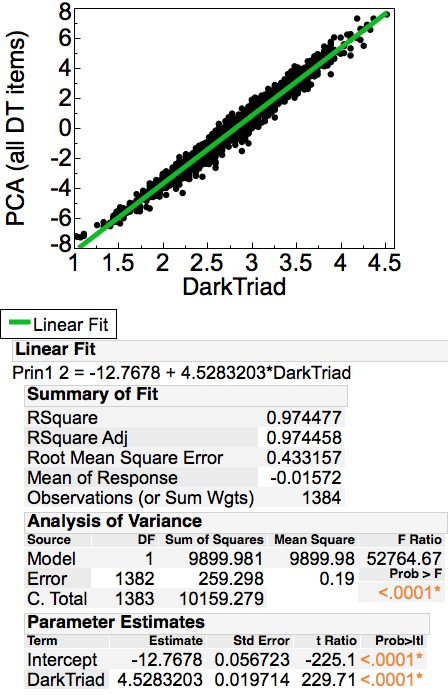


*Cluster Analysis*^.^ We also asked whether participants’ responses on the SD3 clustered into three unique subscales. A post-hoc cluster analysis did not differentiate the items on the SD3 into three trait-based subscales. Cluster 1 contained 10 items from the Psychopathy (*n* = 6) and Machiavellianism subscales (*n* = 4), with the item “Payback needs to be quick and nasty” being most representative. Cluster 2 contained 5 items from the Narcissism subscale, with the item “I know that I am special because everyone keeps telling me so” as most representative. Other items in this scale were: “I have been compared to famous people”, “I insist on getting the respect I deserve”, “Many group activities tend to be dull without me”, “People see me as a natural leader.” Nearly half of the items in the Narcissism scale did not cluster with the items above including: “I am an average person” (reverse coded), “I feel embarrassed if someone compliments me” (reverse coded), “I hate being the center of attention” (reverse coded), “I like getting acquainted with important people.” Clusters 3-6 contained between 2-5 items; cluster 5 contained exclusively items from the psychopathy scale (P7 and P8: “I have never gotten in trouble with the law” (reverse coded) and “I enjoy having sex with people I hardly know”), and cluster 6 contained exclusively items from the machiavellianism scale (“It is not wise to tell your secrets” and “There are things you should hide from other people to preserve your reputation”).


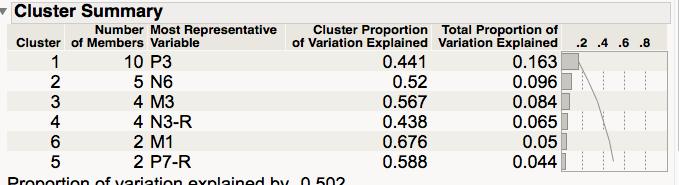


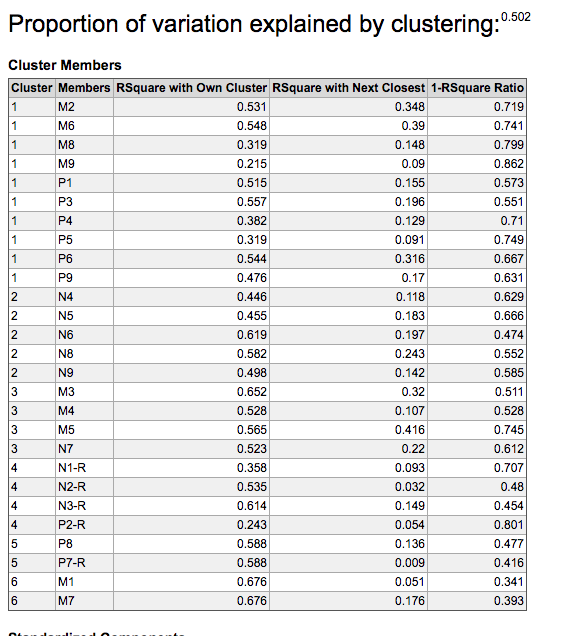


Above is the output for our Cluster Analysis of the Dark Triad measure. M indicates that the item was originally thought to be part of the Machiavellianism subscale, P = Psychopathy, and N = Narcissism. “R” indicates that the item had been reverse coded (see Jones & Paulhus, 2014).

**Multi-Group Confirmatory Factor Analysis.** We also conducted a Multi-Group Confirmatory Factor Analysis (MGCFA). Our initial goal was to test for structural invariance in the Short Dark Triad measure between the US and Indian samples. To do this, we used the R package Lavaan (Rosseel, 2012). First we conducted a confirmatory factor analysis specifying a three factor model and found that the model only had a weak fit to the data (χ2 = 2786.41; DF = 321; CFI = 0.867; gamma hat = .87; RMSEA = 0.085; SRMR = 0.073). Because we failed to find that the model had a good fit to the data, we were unable to proceed with a strong test of structural invariance. Future researchers should further investigate the psychometric properties of the SD3.

**Life history*.*** Past research has found that the Dark Triad traits correspond to a fast life history strategy (Jonason, Icho, & Ireland, 2016). Based on this logic and our hypothesis that the Dark Triad would predict self-maximizing behavior, we asked whether Life History score would also predict defection in the Prisoner’s Dilemma. We found, contrary to our hypothesis, that Life History strategy scores did not predict defection in the Prisoner’s Dilemma (*B* = -.03, *SE* = .054, *p* = .57, OR: 0.97, CI: 0.87, 1.08); additional analyses revealed that life history did not interact with framing. Specifically, in a model predicting Defection from Life History, Social framing, and their interaction, we found that Life History (*B* = -.05, *SE* = .055, *p* = .36, OR: 0.95, CI: 0.85, 1.06) was not a significant predictor of defection while the Social condition was (*B* = -.79, *SE* = .11, *p* < .001, OR: 0.46, CI: 0.37, 0.56) and while trending, there was no significant interaction between Life History and Social/Non-Social conditions (*B* = -.188, *SE* = .11, *p* = .088, OR: 0.83, CI: 0.67, 1.03). Life History was also not a significant predictor of behavior across Gain/Loss frames (*B* = -.04, *SE* = .05, *p* = .49, OR: 0.96, CI: 0.87, 1.07) and there was no interaction (*B* = -.16, *SE* = .11, *p* = .14, OR: 0.85, CI: 0.69, 1.05). We then asked if participants from India (*M* = 0.064) scored higher on the Life History measure than participants from the US (*M* = -0.08). The results of an independent samples Welch *t*-test support this prediction (*t*(1065) = 5.14, *p* < .0001, *d* = .31).

**Empathy*.*** While our primary interest was to understand the relationship between the Dark Triad traits and self-maximizing behavior, we were also interested in understanding whether high levels of empathy predicted cooperative behavior. Empathy is often lacking or inappropriately displayed in those high in the Dark Triad and its subcomponents (Ali, Amorim, & Tomas Chamorro-Premuzic, 2009; Wai & Tiliopoulos, 2012), and has been previously shown to predict cooperative behavior (Eisenberg & Miller, 1987), even in cases where doing so minimizes selfish gain. A chi-squared analysis revealed that those high in Empathy (Defection = 44.4%) were no more likely to Cooperate than those low in Empathy (Defection = 49.4%; *χ*^2^(1) = 1.67, *p* = .197) and a *t*-test comparing Empathy scores between those who Cooperated and Defected found no significant difference in Empathy score between Cooperators (*M* = 61.57) and Defectors (*M* = 60.79; *t*(1344) = 1.72, *p* = .086, *d* = .09). We then constructed a model predicting defection from Empathy (continuous) and found that Empathy was not a significant predictor of behavior in the Prisoner’s Dilemma (*B* = -.093, *SE* = .054, *p* = .085, OR: 0.91, CI: 0.82, 1.01).

When we included both Social/Non-Social Conditions and Empathy as predictors of defection, we found that Empathy was not a significant predictor of defection (*B* = -.09, *SE* = .05, *p* = .088, OR: 0.91, CI: 0.82, 1.01), the Social condition was a significant predictor (*B* = -.79., *SE* = .10, *p* < .001, OR: 0.45, CI: 0.37, 0.56) and that the interaction between Empathy and Social/Nonsocial, while trending, was insignificant (*B* = -.197, *SE* = .11, *p* = .074, OR: .82, CI: 0.66, 1.02). We then predicted defection from a model containing Empathy, Gain/Loss frame, and their interaction, and found that Empathy was not a predictor (*B* = -.09, *SE* = .05, *p* = .114, OR: 0.92, CI: 0.83, 1.02) and there was no interaction between Empathy and framing (*B* = .02, *SE* = .11, *p* = .854, OR: 1.02, CI: 0.82, 1.26). While not directly motivated by any *a priori* hypotheses, we also looked at whether behavior in the Prisoner’s Dilemma was predicted by the two components of the Empathy measure: Cognitive and Affective Empathy. Cognitive Empathy (*B* = -.021, *SE* = .054, *p* = .699, OR: .98, CI: .88, 1.09) was not a significant predictor of defection, while Affective Empathy was (*B* = -.123, *SE* = .054, *p* = .022, OR: .88, CI: .79, .98).

**Nationality**. Next, we tested whether participants from India were more likely to Defect than participants from the United States (US). Previous work (Bone, McAuliffe, & Raihani, 2016) has found that Indian samples can be more competitive in economic games than US samples. However, in our experiment, a Pearson’s Chi-Squared analysis revealed no significant difference in Defection between Indian (Defection: 40.5%) and US participants (Defection: 44.9%; *χ*^2^(1) = 2.13, *p* = .144). While earlier work has not focused primarily on identifying differences in Dark Triad levels across nationalities, there is some reason to believe that the impact of Dark Triad traits on behavior may vary across contexts (Jonason et al., 2017). Consistent with this, we noted that the vast majority of our participants who were high in the Dark Triad traits were from India; while our sample was made up 45% Indian participants, 68% of those participants who were labeled as “High” in Dark Triad were from our Indian sample, while the Indian sample only made up 20% of the participants labeled as “Low” in Dark Triad. On average, participants from India had higher Dark Triad scores (*M* = 3.13) than participants from the US (*M* = 2.58; *t*(1061) = 17.17, *p* < .001, *d =* 1.04). Thus, we next constructed a model, not planned in our preregistration, predicting Defection from Dark Triad (standardized continuous), Country, and their interaction. In this model, Country (*B* = 0.45, *SE =* 0.15*, p* = .002; OR: 1.57, CI: 1.18, 2.09) and Dark Triad (*B* = 0.30, *SE* = 0.12, *p* = .014, OR: 1.35, CI: 1.07, 1.72), but not their interaction (*B* = -0.03, *SE* = 0.15, *p* = .84) predicted defection. However, we believe these data should be treated with caution as we were unable to test for structural invariance

**Exploratory Analyses**

**Sex.** Past research has sometimes found effects of participant Sex on the relationship between Dark Triad and behavior (Jonason, Li, Webster, & Schmitt, 2009). While effects of Sex seem theoretically likely emerge for the Dark Triad traits in cases that pertain to sexual reproduction (i.e. mating strategies), we had no strong predictions about the effect of Sex on the relationship between Dark Triad and defection (and did not plan to analyze sex at all for this reason). This is because our main dependent measure was unrelated to reproductive strategy, and instead related to resource maximization (which should, presumably, be relevant to both men and women). Thus we conducted a post-hoc analysis to assess whether sex interacted with Dark Triad score to predict behavior in the Prisoner’s Dilemma (unplanned in preregistration, and unlisted even as a possible exploratory analysis). In a model predicting Defection from Dark Triad and Sex, we found no effect of sex on Defection (*B* = 0.06, *SE* = 0.12, *p* = .57, OR: 1.07, CI: 0.85, 1.33) and there was no evidence of an interaction between Sex and Dark Triad score (*B* = 0.07, *SE* = 0.11 , *p* = .56, OR: 1.07, CI: 0.86, 1.34). However, it is worth noting that male participants had significantly higher Dark Triad scores (*M* = 3.09) than female participants did (*M* = 2.93; *t*(1042) = 10.1, *p* < .001, *d* = 0.47), as revealed by post-hoc analyses.


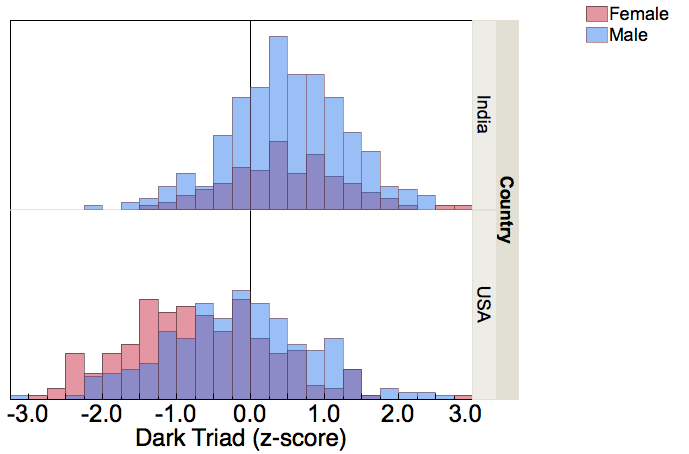


Above: distribution of Dark Triad scores (z-scored) by sex (red = female, blue = male) and country.


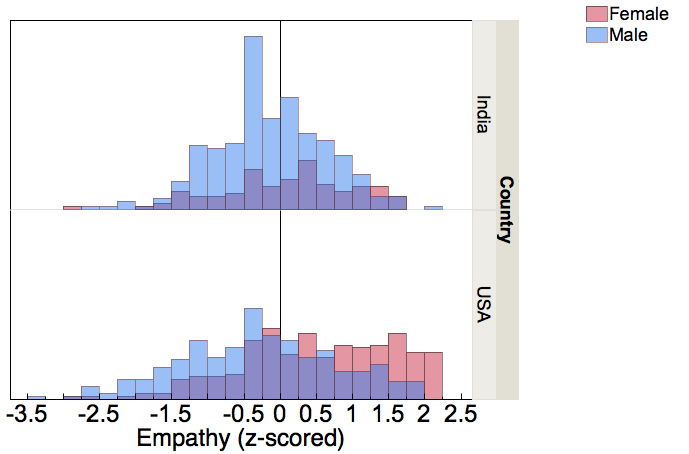


Above: Empathy (z-scored) by sex (red = female, blue = male) and country.

**Country, Condition, Framing, Sex, and Dark Triad**

Because our samples differed so radically in the distribution of Dark Triad scores across nationalities and sex, we conducted one final exploratory analysis (not planned in our preregistration). In this analysis, we constructed a model predicting defection from Condition (Social vs. Nonsocial), Framing (Gain vs. Loss), Country (US vs. India), Sex (Male vs. Female), Dark Triad (continuous standardized) and their interactions. We interpret only the highest level interactions that emerged for any given predictor (full results available: <https://osf.io/knm7u/>).

We found a five-way interaction of Condition, Framing, Sex, Country, and Dark Triad (*B* = 3.18, *SE* = 1.62, *p* = .0492, OR: 24.03, CI: 1.07, 623.03). See below for visualizations of these interactions. Of course, 5-way interactions are nearly impossible to interpret, and the fact that this analysis was unplanned suggests to us that at least some of these effects are likely to be spurious. Still, we present several visualizations below for future researchers to engage with.

Below: Five-way interaction. Shaded is fit confidence. Note that these data are depicted as continuous in these figures, but were, of course, binomial. Note that *n*’s for each cell are listed in the figure; there is a substantial sex imbalance in our Indian population, and no cell contains more than 38 Indian women.

**
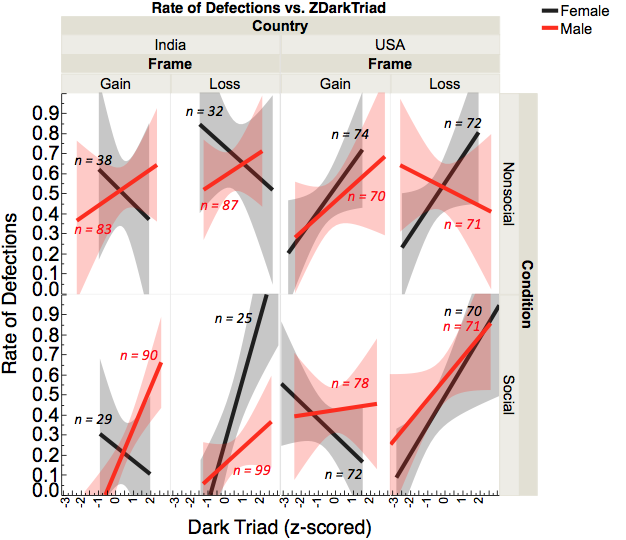
**

**References**

Ali, F., Amorim, I., Chamorro-Premuzic. (2009). Empathy deficits and trait emotional intelligence in psychopathy and Machiavellianism. *Personality and Individual Differences, 47*(7), 758-762.

Copping, L. T., Campbell, A., & Muncer, S. (2014). Psychometrics and life history strategy: The structure and validity of the high K strategy scale. Evolutionary Psychology, 12(1).

Eisenberg, N., Miller, P. A. (1987). The Relation of Empathy to Prosocial and Related Behaviors. *Psychological Bulletin, 101*(1), 91-119.

Hirschfeld, G., & Von Brachel, R. (2014). Multiple-Group confirmatory factor analysis in R-A tutorial in measurement invariance with continuous and ordinal indicators. *Practical Assessment, Research & Evaluation*, *19*(7).

Jonason, P. K., Koenig, B. L., & Tost, J. (2010). Living a Fast Life. *Human Nature*, *21*(4), 428–442.

Jonason, P. K., Icho, A., & Ireland, K. (2016). Resources, Harshness, and Unpredictability
The Socioeconomic Conditions Associated With the Dark Triad Traits. *Evolutionary Psychology, 14*(1), 1-11.

Jones, D. N. & Paulhus D. L. (2014). Introducing the Short Dark Triad (SD3): A Brief Measure of Dark Personality Traits. *Assessment*, *21*, 28-41.

Yves Rosseel (2012). lavaan: An R Package for Structural Equation Modeling. Journal of Statistical Software, 48(2), 1-36. URL <http://www.jstatsoft.org/v48/i02/>.

Wai, M., & Tiliopoulos, N. (2012). The affective and cognitive empathic nature of the dark triad of personality. *Personality and Individual Differences*, *52*(7), 794–799.
